# Nuclear PKC-θ facilitates rapid transcriptional responses in human memory CD4^+^ T cells through p65 and H2B phosphorylation

**DOI:** 10.1242/jcs.181248

**Published:** 2016-06-15

**Authors:** Jasmine Li, Kristine Hardy, Chan Phetsouphanh, Wen Juan Tu, Elissa L. Sutcliffe, Robert McCuaig, Christopher R. Sutton, Anjum Zafar, C. Mee Ling Munier, John J. Zaunders, Yin Xu, Angelo Theodoratos, Abel Tan, Pek Siew Lim, Tobias Knaute, Antonia Masch, Johannes Zerweck, Vedran Brezar, Peter J. Milburn, Jenny Dunn, Marco G. Casarotto, Stephen J. Turner, Nabila Seddiki, Anthony D. Kelleher, Sudha Rao

**Affiliations:** 1 Faculty of Education, Science, Technology & Mathematics, University of Canberra, Canberra, Australian Capital Territory 2617, Australia; 2 Department of Microbiology & Immunology, The Doherty Institute for Infection and Immunity, University of Melbourne, Melbourne, Victoria 3010, Australia; 3 The Kirby Institute, UNSW Australia, Sydney, New South Wales 2052, Australia; 4 The John Curtin School of Medical Research, The Australian National University, Canberra, Australian Capital Territory 0200, Australia; 5 JPT Peptide Technologies Gmbh, Berlin 12489, Germany; 6 Department of Enzymology, Institute of Biochemistry & Biotechnology, Martin-Luther-University Halle-Wittenberg, Halle 06108, Germany; 7 INSERM U955 Eq16 Faculte de medicine Henri Mondor and Universite Paris-Est Creteil/Vaccine Research Institute, Creteil 94010, France

**Keywords:** Chromatin, Memory, PKC-θ, T-cells, Transcription

## Abstract

Memory T cells are characterized by their rapid transcriptional programs upon re-stimulation. This transcriptional memory response is facilitated by permissive chromatin, but exactly how the permissive epigenetic landscape in memory T cells integrates incoming stimulatory signals remains poorly understood. By genome-wide ChIP-sequencing *ex vivo* human CD4^+^ T cells, here, we show that the signaling enzyme, protein kinase C theta (PKC-θ) directly relays stimulatory signals to chromatin by binding to transcriptional-memory-responsive genes to induce transcriptional activation. Flanked by permissive histone modifications, these PKC-enriched regions are significantly enriched with NF-κB motifs in *ex vivo* bulk and vaccinia-responsive human memory CD4^+^ T cells. Within the nucleus, PKC-θ catalytic activity maintains the Ser536 phosphorylation on the p65 subunit of NF-κB (also known as RelA) and can directly influence chromatin accessibility at transcriptional memory genes by regulating H2B deposition through Ser32 phosphorylation. Furthermore, using a cytoplasm-restricted PKC-θ mutant, we highlight that chromatin-anchored PKC-θ integrates activating signals at the chromatin template to elicit transcriptional memory responses in human memory T cells.

## Abstract (precis)

**Summary:** Memory T cells have a rapid transcriptional program upon re-stimulation. Chromatin-anchored PKC-θ integrates activating signals at the chromatin template to elicit this transcriptional memory in T cells.

## INTRODUCTION

As part of the adaptive immune response, memory T and B cells are formed following infection. Their rapid and robust response to re-infection is the basis of immunological memory and vaccination. This rapid transcriptional upregulation or transcriptional memory response of pro-inflammatory genes is in part epigenetically regulated, with immediate gene expression attributed to permissive chromatin modifications such that gene loci such as *IL2* and *IFNG* are characterized by increased enrichment of acetylated lysine 9 (H3K9ac) and tri-methylated lysine 4 on H3 (H3K4me3) and demethylated CpG islands (Barski et al., 2007; Denton et al., 2011; Kersh et al., 2006; Murayama et al., 2006; Russ et al., 2014). However, the molecular basis of how the permissive epigenetic landscape integrates incoming signals to induce transcriptional memory remains elusive.

The serine/threonine-specific kinase protein kinase C theta (PKC-θ) plays diverse roles in immune cells (Kong and Altman, 2013). T cell activation recruits PKC-θ to the immunological synapse to initiate the formation of the CARMA–BCL10–MALT (CBM) signaling complex and nuclear translocation of NF-κB family members for transcriptional programs necessary for T cell survival, proliferation and homeostasis (Smale, 2012; Smith-Garvin et al., 2009). The absence of PKC-θ impairs nuclear translocation of activator protein 1 (AP-1) and NF-κB in T cells (Sun et al., 2000) and compromises antigen-specific T_H_1 and T_H_2 cell proliferation and qualitative responses in autoimmune, allergic and helminthic infection models (Healy et al., 2006; Manicassamy et al., 2006; Marsland et al., 2004; Salek-Ardakani et al., 2005). In terms of immunological memory, PKC-θ is required for lymphocytic choriomeningitis virus (LCMV) antigen recall in CD8^+^ T cells *in vitro* (Marsland and Kopf, 2008; Marsland et al., 2005), and even delayed PKC-θ signaling severely impedes memory T cell development (Teixeiro et al., 2009).

All PKC family members have the ability to translocate to the nucleus through a nuclear localization signal (NLS) (DeVries et al., 2002; Sutcliffe et al., 2012). Despite the importance of PKC-θ in T cell development, how its nuclear activity facilitates transcriptional memory responses is still largely unknown. To this end, we used genome-wide chromatin immunoprecipitation (ChIP)-sequencing to show that nuclear PKC-θ directly localizes to permissive regions enriched for nuclear factor κB (NF-κB)-binding sites in *ex-vivo*-derived human memory CD4^+^ T cells and transcriptional-memory-responsive Jurkat T cells. Upon T cell activation, PKC-θ not only still bound to the p65 subunit (also known as RelA) of NF-κB through Ser536 phosphorylation but also sustained chromatin accessibility by specifically phosphorylating Ser32 of the core nucleosomal component histone H2B. Hence, nuclear PKC-θ directly delivers T cell activating signals to the chromatin template for rapid transcriptional memory responses in human memory CD4^+^ T cells.

## RESULTS

### Rapid transcriptional responses in memory CD4^+^ T cells depends on PKC-θ signaling

**Fig. 1.**
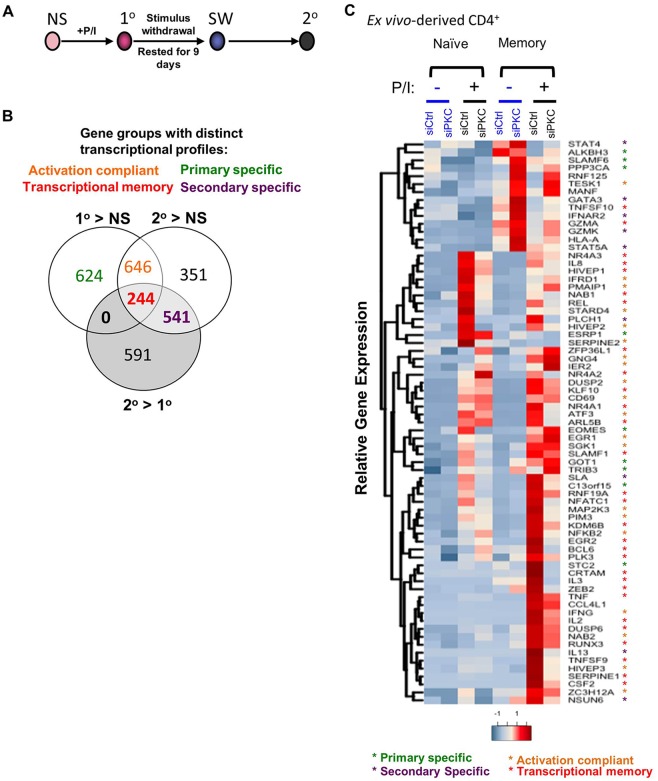
**PKC-θ signaling and rapid transcriptional responses in memory CD4^+^ T cells.** (A) A schematic of the *in vitro* transcriptional memory Jurkat T cell model: non-stimulated (NS) Jurkat T cells were activated with PMA and Ca^2+^ ionophore (+P/I, denoted 1°) and then subjected to stimulus withdrawal (SW) for 9 days before re-stimulation (2°). (B) Venn diagram showing the number of genes grouped by their distinct transcriptional profiles in the Jurkat model. These profiles are for the primary-specific, activation-compliant, transcriptional-memory-responsive and secondary-specific groups. (C) Heatmap representation of inducible gene expression in naïve and memory CD4^+^ T cells treated with PKC-θ siRNA (siPKC) with and without PMA and Ca^2+^ ionophore. Gene expression normalized to *GAPDH* is represented as *z*-scores (mean, *n*=2). The colors of the asterisk correspond to the gene groups shown in Fig. 1B. siCtrl, control siRNA.

To investigate the importance of PKC-θ signaling in transcriptional memory responses, we devised an *in vitro* transcriptional memory model in which non-stimulated Jurkat T cells were stimulated with the PKC pathway inducers PMA and Ca^2+^ ionophore for 4 h (denoted as the primary stimulation). This was followed by stimulus withdrawal and re-stimulation (denoted as the secondary stimulation) (Fig. 1A). Whole-transcriptomic analysis showed that a majority (but not all) stimulation-induced expression changes were reversible following stimulus removal, with expression more variable during re-stimulation (Fig. S1A). Compared to in non-stimulated cells, Gene Set Enrichment Analysis (GSEA) showed that highly expressed genes in cells subjected to stimulus withdrawal were characteristically associated with effector memory (T_EM_) and central memory (T_CM_) T cells. Similarly, more memory-cell-associated genes were upregulated in the re-stimulated (secondary) Jurkat T cells compared to cells activated by the primary stimulation (Table S1; Abbas et al., 2005, 2009; Luckey et al., 2006; Wherry et al., 2007).

We next compared gene induction after the primary and secondary stimulations to identify distinct transcriptional gene subsets, such as genes whose expression was upregulated after only the primary stimulation (primary specific) or only the secondary stimulation (secondary specific) or that remain unchanged between the primary and secondary stimulation (activation compliant). Importantly, a group of genes highly and rapidly expressed on re-stimulation was also identified (transcriptional memory) (Fig. 1B). Despite multiple cell divisions (Fig. S1B), an experience of primary activation resulted in greater and faster *IL2*, *TNF* and *IFNG* transcription during secondary activation, but this did not occur for the early activation marker *CD69* (Fig. S1C). This rapid expression is characteristic of polyfunctional memory CD4^+^ T cells, such that IL-2 facilitates CD4^+^ and CD8^+^ T cell expansion whereas TNF-α (also known as TNF) and IFN-γ are crucial for effector functions (Seder et al., 2008). Furthermore, protective vaccination therapy has been shown to induce a higher frequency of IFN-γ-, TNF-α- and IL-2-producing CD4^+^ T cells (Darrah et al., 2007). Therefore, understanding the transcriptional regulation of these transcriptional-memory-responsive genes is clearly relevant to the function of memory T cells.

Having identified these gene groups with distinct transcriptional profiles in the Jurkat T cell model, we investigated the role of PKC-θ in the upregulation of these genes in bulk-sorted naïve and memory CD4^+^ T cells (Fig. 1C). We firstly validated that PKC-θ small interfering RNA (siRNA) was effective at specifically knocking down PKC-θ by ∼50% without affecting the expression of PKC-α and PKC-δ (Fig. S1D). Comparisons of naïve and memory CD4^+^ T cells transfected with the control siRNA demonstrated that memory T cells had an overall higher transcriptional induction than naïve T cells for the genes analyzed. However, PKC-θ knockdown selectively inhibited upregulation of immune-regulatory genes by 29–91% in activated memory CD4^+^ T cells including *IL2*, *IFNG* and *TNF*, other chemokines like *IL8* and *TNFSF9*, and transcription factors such as *NFKB2*, *REL* and the NR4A gene family (*NR4A1, NR4A2* and *NR4A3*) (Fig. 1C). In contrast, the downregulation of PKC-θ did not affect the inducible transcription of the early activation marker *CD69.* Its transcription was consistently upregulated in both the control- and PKC-θ-siRNA-treated memory CD4^+^ T cells activated with the PMA and Ca^2+^ ionophore. This indicates that PKC-θ signaling is directly responsible for a distinct gene expression program in differentiated human memory CD4^+^ T cells.

### Genome-wide distribution of PKC-θ in memory T cell development

**Fig. 2.**
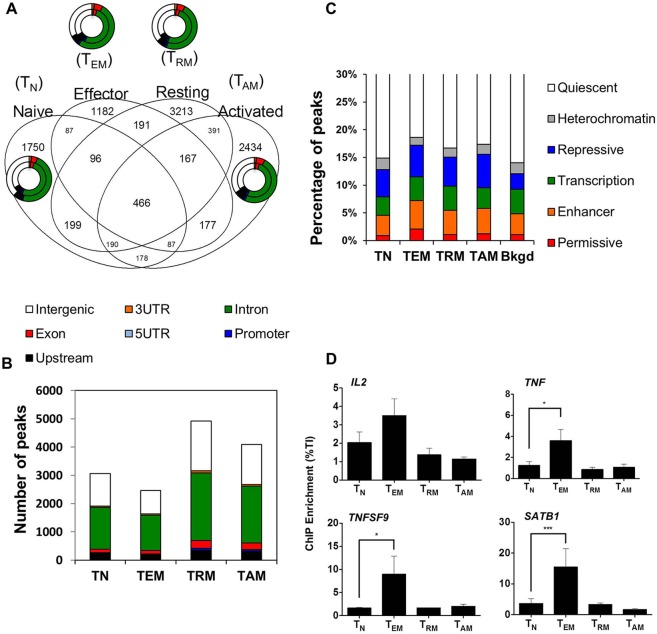
**Distribution of chromatinized PKC-θ in memory CD4^+^ T cells.** (A) Overlapping PKC-θ-bound regions in *ex-vivo*-derived naïve (T_N_), effector (T_EM_), resting (T_RM_) and activated memory (T_AM_) CD4^+^ T cells. Graphs show genomic annotation of unique peaks (outside ring) compared to the peaks common to all subsets (inside ring). (B) PKC-θ peaks in memory CD4^+^ T cell subsets categorized into intergenic, 3′ untranslated region (UTR), intron, exon, 5′ UTR, promoter and upstream regions according to the nearest ENSEMBL gene, see corresponding colors in A. (C) The percentage of PKC-θ peaks in memory CD4^+^ T cell subsets and genomic background (Bkgd) annotated according to their chromatin state segmentation: permissive (H3K4me3, H3K27ac and H2K9ac), transcription (H3K36me3), enhancer (H3K4me1, and to different degrees H3K27ac or H3K9ac), repressed (H3K27me3 and/or H3K9me3), heterochromatin (H3K9me3) and quiescent (associated with low levels of histone marks). (D) Chromatin immunoprecipitation (ChIP-PCR) analysis on PKC-θ at the proximal promoters of the transcriptional-memory-responsive genes: *IL2*, *TNF* and *TNFSF9* and an intronic region of *SATB1*. ChIP enrichment is shown as a percentage relative to the total input (TI) with background subtraction (mean±s.e.m., *n*=3). *P≤0.05, ***P≤0.001 (one-tailed Student's *t*-test).

We previously reported the recruitment of PKC-θ to gene promoters in Jurkat T cells (Sutcliffe et al., 2011) and because we showed that PKC-θ is necessary for inducible gene expression in primary human memory CD4^+^ T cells (Fig. 1C), we next assessed the genome-wide distribution of chromatin-tethered PKC-θ in *ex vivo* primary human CD4^+^ T cells. *Ex-vivo*-derived T cells were sorted into naïve (T_N_, CD45RO^−^ CD62L^+^ CD127^hi^ CD25^−^) and memory CD4^+^ T cell subsets [effector memory (T_EM_, CD45RO^+^ CD62L^+/−^ CD127^lo^ CD25^−^), resting memory (T_RM_, CD45RO^+^ CD62L^+/−^ CD127^hi^ CD25^−^) and activated memory (T_AM_, CD45RO^+^ CD62L^+^ CD127^hi^ CD25^+^)], where hi denotes high levels of expression, lo denotes low levels of expression, and +/− either positive or negative expression (Fig. S1E). Sequencing chromatin-immunoprecipitated DNA (ChIP-sequencing) pooled from six individually validated ChIPs generated 2,547,999–4,051,661 uniquely mapped reads. A proportion of PKC-θ binding was common to both naïve and memory T cells. However, the majority of binding was cell type specific, with the most PKC-θ peaks detected in T_RM_ cells followed by T_AM_ cells (Fig. 2A), possibly reflecting chromatin remodeling differences in these T cell subsets. PKC-θ binding was distributed between 3′UTRs and gene promoters, with over half the peaks occurring in introns in all T cell subsets (Fig. 2B).

To further profile the chromatin landscape close to PKC-θ binding, all PKC-θ-binding regions were annotated according to their chromatin state segmentation in CD4^+^ T memory cells using RoadMap data (Hoffman et al., 2013) (Fig. 2C). Most PKC-θ was localized to quiescent regions. However, up to 10% of PKC-θ was tethered to permissive, transcriptionally active and enhancer regions. When cross-referenced to primary T cell microarray data, PKC-θ bound to 217, 122, and 144 transcriptionally upregulated genes in T_EM_, T_AM_ or both T_EM_ and T_AM_ cells in the *ex vivo* primary human memory model (E-MEXP-2578; Fig. S1F–J), and 223, 145 and 316 upregulated genes in T_RM_, T_AM_ or both T_RM_ and T_AM_ cells compared to naïve cells in another vaccinia-responsive memory CD4^+^ T cell model (Fig. S2A; Fig. S2B–F). PKC-θ localized to enhancers in intronic regions of highly transcribed genes, e.g. *NFIC*, *SLAMF7*, *YWHAH*, *RUNX2* and *HIVEP3*, in *ex vivo*-bulk and vaccinia-responsive memory CD4^+^ T cells (Figs S1G,H, S2C,D), some of which represent transcription factors known to shape T cell development. This is the first report of the genome-wide distribution of PKC-θ in naïve and memory CD4^+^ T cells to include promoter and intronic enhancer regulatory elements associated with transcriptional upregulation. In order to confirm PKC-θ binding in human CD4^+^ T cells, we carried out ChIP followed by quantitative real-time PCR (ChIP-qPCR) using a pan-PKC-θ antibody and an antibody specifically against the phosphorylated serine 676 (Ser676p) of PKC-θ, an inducible modification indicative of an enzymatically active PKC-θ. We detected a significantly higher amount of pan-PKC-θ and PKC-θ Ser676p binding at the promoters of *IL2*, *TNF*, *TNFSF9* and *SATB1* in T_EM_ compared to T_N_ CD4^+^ T cells (Fig. 2D; Fig. S2H). This significant enrichment of PKC-θ S676 demonstrates that an enzymatically active form of PKC-θ is docked at transcriptionally active gene loci in T_EM_ cells.

Further to our investigation, we examined PKC-θ binding in the human vaccinia-responsive memory model. Given that the expression of granzyme B (*GZMB*) and K (*GZMK*) is known in vaccinia-responsive memory CD8^+^ T cell (Nowacki et al., 2007) and memory CD4^+^ T cell effectors (C.M.L.M. and J.J.Z., unpublished observations), respectively, we investigated the recruitment of PKC-θ at these promoters. Despite the aberrant upregulation of *GZMK* transcription in bulk memory CD4^+^ T cells with PKC-θ knockdown (Fig. 1C), ChIP-qPCR detected increasing but statistically non-significant PKC-θ enrichment across *GZMB* and *GZMK* promoters in vaccinia-responsive memory CD4^+^ T cells (Fig. S2I). The discrepancy between these two results could potentially indicate the requirement of a differential regulation in PKC-θ signaling between the two human CD4^+^ memory models. Overall, these findings suggest that PKC-θ couples to the chromatin structure at the gene promoter and intronic enhancer regulatory elements in bulk and antigen-specific memory CD4^+^ T cells.

### Stimulation-induced PKC-θ recruitment to transcriptional memory-responsive genes

**Fig. 3.**
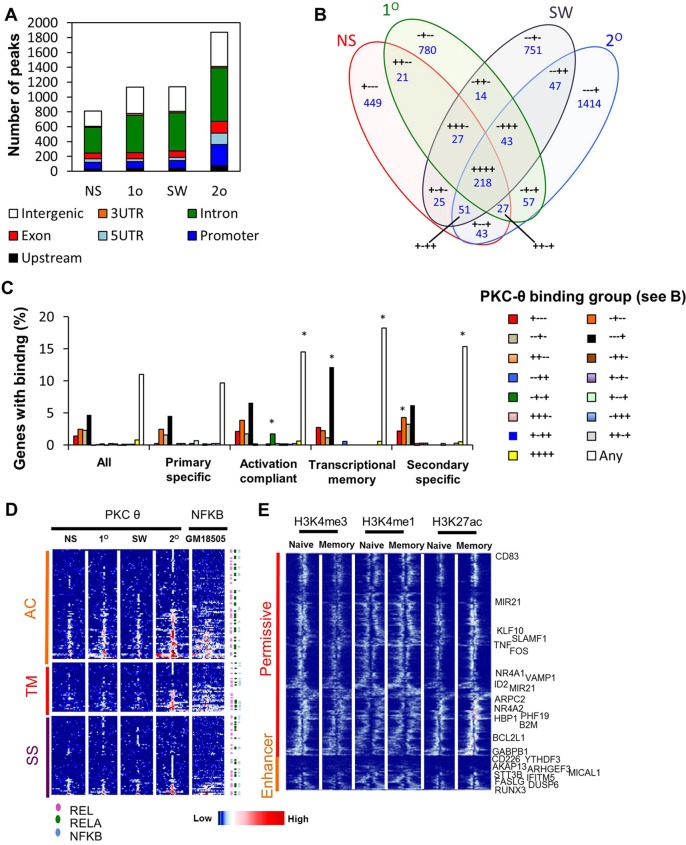
**Inducible recruitment of nuclear PKC-θ in transcriptional memory-responsive Jurkat T cells.** (A) PKC-θ ChIP-seq peaks in non-stimulated (NS) Jurkat T cells, and cells after primary stimulation (1°), stimulus withdrawal (SW), and secondary stimulation (2°) categorized into intergenic, 3′ untranslated region (UTR), intron, exon, 5′ UTR, promoter, and upstream regions as per their nearest ENSEMBL gene. (B) Overlap of ChIP-seq peaks in the different treatments. ‘+’ indicates PKC-θ peak present in treatment, the treatments are listed in order of non-stimulated, primary, stimulus withdrawal and secondary. (C) Proportion of genes from expression groups (see Fig. 1B), with PKC-θ binding in any treatment or in the treatment groups specified in B. *P≤0.05 (Fisher’s exact test against the proportion of all genes on the array). (D) Heatmap representation of PKC-θ enrichment levels for genes in the activation compliant (AC), transcriptional memory (TM) and secondary-specific (SS) groups. p65 ChIP-seq from GM18505 cells (GSM935282) is also shown. Sequencing tags are binned by 100 bp and shown ±3000 bp from the center of PKC-θ binding. NF-κB JASPAR motifs occurring within ±250 bp of the region center are annotated. (E) Overlap of PKC-θ binding in permissive and enhancer regions (re-stimulated Jurkat T cells, 2°) with previously published data on H3K4me3 (GSM772852) and H3K4me1 and H3K27me3 (GSE43119) enrichment in naive and memory CD4^+^ cells. Sequencing tags are binned by 100 bp and shown ±3000 bp from the center of PKC-θ binding. Binding near transcriptional memory and secondary-specific genes are labeled.

To assess PKC-θ recruitment in a stimulus-dependent manner, PKC-θ binding was profiled in Jurkat T cells that were unstimulated, and those subjected to a primary stimulation, after stimulus withdrawal and secondary re-stimulation using ChIP-seq, which generated 6,754,720–13,423,192 non-duplicate reads and 811–1872 enriched regions (Fig. 3A). Here, chromatin tethering of PKC-θ was stimulus-dependent with PKC-θ-binding changes being reversible upon resting. Importantly, PKC-θ recruitment differed between primary and secondary activated cells, with an increased number of promoters and 5′UTR having recruitment in secondary activated cells (Fig. 3A,B; Fig. S3A). This was exemplified by a significant amount of PKC-θ enrichment throughout the gene body of *TNF* during secondary re-stimulation (Fig. S3B). Comparison between PKC-θ binding and gene expression showed that many genes with PKC-θ bound in the different treatments did not change expression (Fig. S3C). Upon segregating gene expression based on the location of PKC-θ recruitment, we observed that genes with PKC-θ binding at the promoter, 5′UTR, exon or 3′UTR showed a higher median expression than all genes represented on the array (Fig. S3D), and 19–36% of these regions had general permissive chromatin marks in lymphocytes (Roadmap data, Fig. S3E). Furthermore, genes with the activation compliant, transcriptional memory and secondary-specific transcriptional profile were significantly enriched for PKC-θ binding, with the activation compliant group having binding upon primary and secondary stimulation, and transcriptional-memory-responsive genes showing binding predominantly after re-stimulation (Fig. 3C,D, Fig. S3F).

As with primary T cells, a large proportion of annotated regions (∼40%) across all treatments were introns, with a significant proportion representing 5′UTRs and promoters [up to –1 kb from the transcription start site (TSS)], increasing to 24% of total in secondary activated cells (Fig. 3A). PKC-θ-targeted regions were flanked by permissive or enhancer marks such as H3K4me3, H3K4me1 and H3K27ac (Fig. 3E) in human naïve and memory CD4^+^ T cells (data from Schmidl et al., 2014), many of which were near transcriptional memory or memory-specific genes. Furthermore, many PKC-θ-bound genes with increased expression in secondary activated Jurkat T cells also showed upregulated expression in both activated memory and vaccinia-responsive CD4^+^ T cells (Fig. S3F).

To assess functional PKC-θ binding, we used the cis-regulatory element annotation system (CEAS) to investigate the conservation scores of PKC-θ-bound regions derived from the Jurkat T cell model (Ji et al., 2006). As expected from their promoter-biased distribution, the average conservation score for PKC-θ-targeted regions in Jurkat T cells displayed a good degree of conservation (Fig. S3G). Over-represented DNA motifs were identified in PKC-θ bound regions in secondary activated cells (Tables S2, S3). Consistent with our previous findings (Sutcliffe et al., 2012; Zafar et al., 2014), an NF-κB-like motif and other motifs such as NRF, NFY and SP1 were enriched (HOMER; Table S2). When promoter-focused predictions were limited to motifs with higher ‘activity’ in the primary and secondary expression profiles (ISMARA; Table S3), only the NF-κB motif remained associated with higher PKC-θ binding and gene expression. Interrogation of publicly available ChIP-seq data (GM18505 cells) confirmed that p65 bound close to PKC-θ for upregulated genes (Fig. 3D), suggesting that NF-κB family members, and specifically p65, could cooperate with PKC-θ at these loci. DNA-binding motifs for NF-κB are located within 0.25 kb of the majority of PKC-θ-binding sites associated with inducible genes (Fig. 3D). GSEA supported this hypothesis, because genes expressed in cells subjected to stimulus withdrawal were enriched for NF-κB regulation processes compared to non-stimulated cells (Table S4), and secondary activated cells were enriched for genes with NF-κB-binding motifs (Table S5). There was also a significant enrichment of genes associated with inhibitory κB (IκB) kinase (IKK) or NF-κB-regulation in the primary human and vaccinia-responsive memory models (Table S4), particularly enrichment of genes with the binding motifs for the NF-κB family member REL in vaccinia-responsive T_RM_ and T_EM_ cells (Table S5). This suggests a potential synergistic co-operation between PKC-θ and NF-κB signaling, with transcription of NF-κB pathway genes actively directing nuclear PKC-θ to transcriptional memory gene promoters.

### Increased p65 nuclear translocation in memory CD4^+^ T cells

**Fig. 4.**
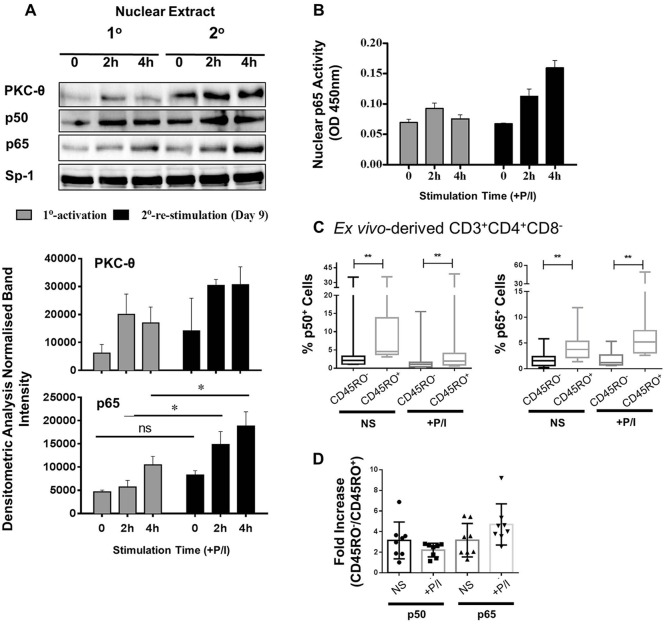
**Colocalization of PKC-θ and NF-κB in T cells.** (A) Representative immunoblots of PKC-θ, p50 and p65 protein levels in the nuclear extracts of activated Jurkat T cells (0, 2, 4 h) during primary (1°), and secondary (2°) stimulation with Sp1 as the loading control (representative graph of three independent repeats). The corresponding p65 and PKC-θ (normalized to Sp-1) densitometry is shown below (mean±s.e.m., *n*=3). *P≤0.05; ns, not significant (two-way ANOVA). (B) NF-κB (p65) activity detected in nuclear extracts from Jurkat T cells during day 0, primary, and day 9, secondary, activation (0, 2 and 4 h) with PMA and Ca^2+^ ionophore (+P/I). Representative graph of three independent replicates, error bars show s.e.m. of technical replicates. (C) The percentage of CD45RO^−^ (naïve) and CD45RO^+^ (memory) *ex-vivo*-derived human CD4^+^ T cells with p50 and p65 staining as detected by using flow cytometry with (+P/I) or without (NS) PMA and Ca^2+^ ionophore (median±quartiles, *n*=6). The whiskers represent the first and fourth quartiles. **P≤0.01 (Wilcoxon matched-pairs signed rank test was used to compare groups). (D) p50 and p65 staining levels were calculated as fold-changes between human CD45RO^−^ (naïve) and CD45RO^+^ (memory) T cells with (+P/I) or without (NS) PMA and Ca^2+^ ionophore (mean±s.e.m., *n*=6).

To quantify the relationship between nuclear translocation and gene-specific recruitment of PKC-θ and p65, nuclear p50 and p65 protein expression was examined in primary and secondary activated Jurkat T cells. Despite the basal expression of nuclear PKC-θ in non-stimulated cells, PKC-θ expression increased after primary and secondary activation. This was particularly associated with p65 nuclear translocation (Fig. 4A) and activity in secondary activated cells (Fig. 4B). In contrast, the levels of the p50 NF-κB subunit remained constant (Fig. 4A). ChIP-qPCR also demonstrated that the increase of PKC-θ enrichment in the secondary activated Jurkat T cells was colocalized with p65 and Pol II at the two-well characterized p65-binding promoters (Himes et al., 1993) *IL2* and *TNF*, as well as at *TNFSF9* and *SATB1* (Fig. S4A), but not at a PKC-θ non-binding region in *SMAD3* (Fig. S4B). We then sorted bulk naïve and memory human CD4^+^ T cells to show that higher levels of p65 and p50 were detected in resting human memory (CD45RO^+^) CD4^+^ T cells than naïve cells (CD45RO^−^), with a further increase demonstrable upon activation with PMA and Ca^2+^ ionophore (Fig. 4C,D). ChIP-PCR analysis showed a similar, but stimulus-dependent, increase in p65 enrichment at the *TNF* promoter in activated memory CD4^+^ T cells (Fig. S4C). Therefore, we found that PKC-θ and p65 recruitment was more effective in both transcriptional-memory-responsive Jurkat T cells and primary human CD4^+^ memory T cells.

### Cytoplasmic versus nuclear PKC-θ

**Fig. 5.**
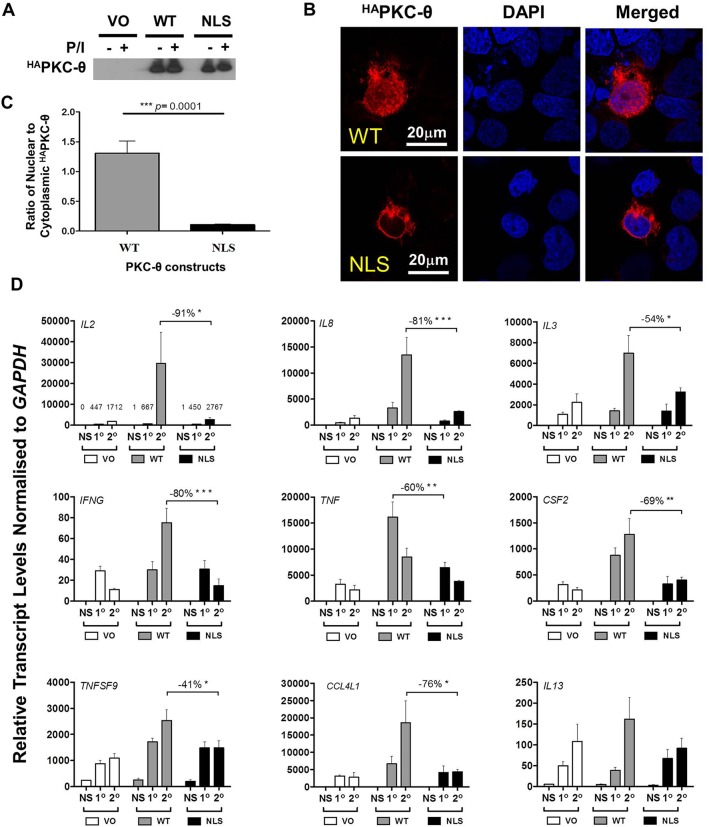
**Direct regulation of gene expression by nuclear PKC-θ.** (A) Representative western blot of HA-tagged PKC-θ protein (^HA^PKC-θ) levels in non-stimulated (NS) and PMA and Ca^2+^ ionophore (P/I)-activated Jurkat T cells transfected with vector only (VO), wild-type PKC-θ plasmid (WT) or cytoplasmic-restricted PKC-θ mutant (NLS) plasmids. (B) Representative confocal microscopy images showing subcellular localization of PKC-θ in Jurkat T cells transfected with WT or cytoplasm-restricted PKC-θ (NLS) plasmids. (C) Quantification of microscopy to show the ratio of nuclear to cytoplasmic-located HA-tagged PKC-θ in Jurkat T cells transfected with WT or cytoplasm-restricted PKC-θ (NLS) plasmids (mean±s.e.m., *n*=3 repeats). ***P<0.0001 (Mann–Whitney test). (D) Gene expression of transcriptional-memory-responsive genes in Jurkat T cells transfected with vector only (VO), wild-type (WT) and cytoplasm-restricted PKC-θ mutant plasmids (NLS) (mean±s.e.m., *n*=3) with the percentage inhibition calculated between the WT and NLS during primary (1°) activation for *TNF* and secondary (2°) activation for *IL2*, *IL8*, *IL3*, *IFNG*, *CSF2*, *CCL4L1* and *TNFSF9*. NS, non-stimulated cells. *P≤0.05, **P≤0.01 and ***P≤0.001 (two-way ANOVA).

To distinguish the cytoplasmic and nuclear roles of PKC-θ in transcriptional memory responses, a HA-tagged wild-type PKC-θ (^HA^PKC or WT) or a PKC-θ-NLS mutant (NLS-PKC) was expressed in Jurkat T cells. Although the total ^HA^PKC-θ protein expression was similar for all PKC-θ constructs (Fig. 5A), a significant proportion of NLS-PKC-θ was restricted to the cytoplasm (Fig. 5B,C). Having a mutated NLS significantly reduced the expression of certain effector genes, such as *TNF*, during primary activation, but this inhibition was particularly evident during re-stimulation for *IL2*, *IL8*, *IL3*, *CSF2*, *CCL4L1*, *IFNG* and *TNFSF9* (Fig. 5D). Using this novel PKC-NLS mutant, we showed that nuclear translocation of PKC-θ is required for optimal transcriptional memory responses.

### p65 Ser536 phosphorylation by nuclear PKC-θ

**Fig. 6.**
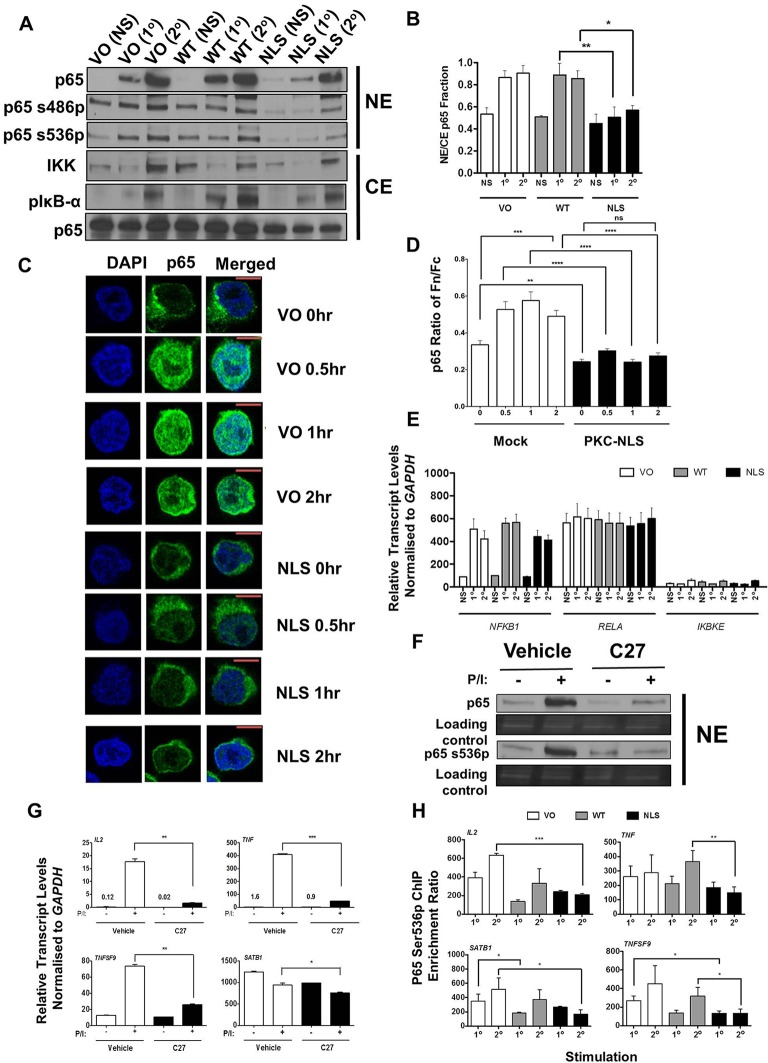
**p65 Ser536 phosphorylation by nuclear PKC-θ.** (A) Immunoblotting of p65, p65 phosphorylated at Ser486 (p65s486p) and Ser536 (p65s536p) in the nuclear extract (NE), and IKK, phosphorylated IκB-α on Ser32 or Ser36 (pIκB-α) and total p65 levels in the cytoplasmic extract (CE) in non-stimulated (NS) Jurkat T cells, and cells after primary (1°) and secondary (2°) stimulations transfected with vector only (VO), wild-type PKC-θ plasmid (WT) or cytoplasmic-restricted PKC-θ mutant (NLS) plasmids. Representative blot of three independent repeats (also see Fig. S4D,E). (B) Nuclear-to-cytoplasmic ratios (NE/CE) of p65 protein levels immunoblotted in nuclear versus cytoplasmic fractions was calculated for the cells described in A (mean±s.e.m., *n*=3). *P≤0.05, **P≤0.01 (two-way ANOVA). (C) Confocal microscopy of p65 nuclear localization during activation with PMA and Ca^2+^ ionophore for 0, 0.5, 1 and 2 h in Jurkat T cells transfected with vector only (VO) or the cytoplasm-restricted PKC-θ (NLS) mutant plasmids. Cells were stained with DAPI and anti-p65 antibodies. Representative images of these constructs. Scale bars: 20 μm. (D) Nuclear translocation of p65 (Fn/Fc) expressed as a ratio of nuclear (Fn) and cytoplasmic (Fc) fluorescence with background subtraction (mean±s.e.m., *n*>20 cells). **P≤0.01; ***P≤0.001; ****P<0.0001; ns, not significant (Mann–Whitney test). (E) Gene expression of p50 (*NFKB1*), p65 (*RELA*), and (*IKBKE*) in non-stimulated (NS) Jurkat T cells, and cells after primary (1°) and secondary (2°) stimulations transfected with VO, WT and NLS plasmids (mean±s.e.m., *n*=2). (F) Immunoblotting of p65 and p65 phosphorylated serine 536 (p65 Ser536p) levels in nuclear extract from DMSO or 1 μM C27-treated Jurkat T cells with or without PMA and Ca^2+^ ionophore (P/I) (*n*=6). (G) *GAPDH*-normalized gene expression of *IL2*, *TNF*, *TNFSF9* and *SATB1* in the vehicle-control or 1 μM C27-treated Jurkat T cells with or without PMA and Ca^2+^ ionophore (P/I) (mean±s.e.m., *n*=3). *P≤0.05, **P≤0.01, ***P≤0.001 (one-tailed Student's *t*-test). (H) ChIP-PCR analysis of p65 Ser536p at the promoters of *IL2*, *TNF*, *TNFSF9* and an intronic region of *SATB1* in Jurkat T cells expressing VO, WT and NLS plasmids after primary (1°) and secondary (2°) stimulations. ChIP enrichment ratio relative to the no-antibody control is shown (mean±s.e.m., *n*=3). *P≤0.05, **P≤0.01, ***P≤0.001 (one-tailed Student's *t*-test).

Having established that nuclear PKC-θ is important for inducible gene induction, we sought to establish how PKC-θ regulates p65. We investigated Ser486 and Ser536 phosphorylation because these events are central to canonical NF-κB signaling (Hochrainer et al., 2013). As anticipated, the overall nuclear p65 translocation increase in Jurkat T cells overexpressing the vector only and wild-type PKC-θ (WT) plasmids was greater during secondary activation than upon primary activation. In contrast, p65 translocation was inhibited in NLS-PKC mutant cells (Fig. 6A,B; Fig. S4D,E). Despite the increase of both nuclear p65 Ser486p and Ser536p in stimulated cells, the NLS-PKC mutation selectively reduced the levels of nuclear p65 and p65 Ser536p after primary and secondary activation compared to controls, without affecting Ser486p levels (Fig. S4E). This reduced p65 and Ser536p corresponded to gene-specific transcriptional inhibition shown in Fig. 5D. When nuclear-to-cytoplasmic ratios were considered, nuclear p65 increased during primary and secondary activation in vector only and WT cells but it was abolished in the NLS mutant (Fig. 6C,D), suggesting that a lack of chromatinized PKC-θ prevents nuclear p65 retention. Immunofluorescence showed that nuclear p65 was significantly reduced in PKC-NLS-transfected cells compared to the control during primary and secondary activation (Fig. S4F,G). Furthermore, reduced p65 nuclear translocation persisted over time (0.5–2 h), with no p65 shuttling detected above non-stimulated levels in the NLS-PKC mutant (Fig. 6C,D). This defective NF-κB signaling in the PKC-NLS mutant was not simply due to deregulated NF-κB production, because IKK (*IKBKE*), p50 (*NFKB1*) and p65 (*RELA*) transcription were the same in vector only, WT, and NLS-PKC cells (Fig. 6E). These data led us to hypothesize that one function of nuclear PKC-θ is to maintain p65 nuclear retention through Ser536 phosphorylation in a signal-dependent manner.

To address this hypothesis, the ATP-competitive PKC-θ inhibitor compound 27 (C27) was used to assess the transcription and epigenetic status of PKC-θ-targeted genes in Jurkat T cells (Jimenez et al., 2013). Indeed, C27 exerted an overall inhibitory effect on the nuclear expression of p65 (Fig. 6F) and significantly reduced the transcription of PKC-θ bound genes: *IL2*, *TNF*, *TNFSF9* and *SATB1* (Fig. 6G). We next compared enrichment of p65 Ser536p during primary and secondary activation in Jurkat T cells overexpressing the wild-type or NLS-PKC. Relative to either the vector only or WT controls, ChIP-qPCR detected a significant loss of p65 Ser536p at *IL2*, *TNF*, *TNFSF9* and *SATB1* genes in the NLS-PKC mutant during secondary activation (Fig. 6H). These results confirmed that nuclear PKC-θ catalytic activity is important for p65 Ser536 phosphorylation and its tethering to transcriptional-memory-responsive gene promoters in T cells.

### PKC-θ-mediated chromatin accessibility is modulated through H2B phosphorylation

**Fig. 7.**
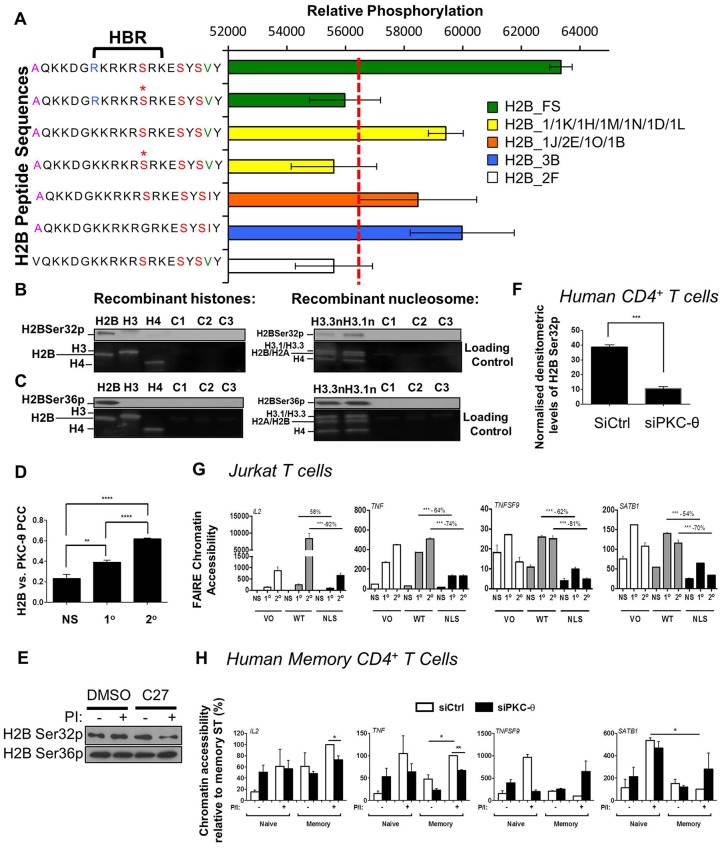
**Identification of phosphorylated residues on histone H2B.** (A) PKC-θ-mediated phosphorylation signals on H2B:21 (residues 21–40 derived from H2B) were detected by PKC-θ microarray profiling. The mean phosphorylation is shown (±s.d.). * denotes a pre-phosphorylated serine and the dotted red line is the background threshold (57500). The location of the histone H2B repression domain (HBR) is marked. (B) PKC-θ phosphorylates H2B Ser32. An *in vitro* kinase assay was performed by incubating active PKC-θ with either recombinant histones H2B, H3 or H4 or recombinant nucleosomes containing H3.1 or H3.3. Phosphorylated proteins were resolved by SDS-PAGE followed by western blotting for H2B phosphorylation. Negative controls include C1 (no ATP addition), C2 (no PKC addition), and C3 (incubation with PKC-μ). A representative blot of three experiments is shown. (C) PKC-θ phosphorylates H2B Ser36, as assessed by the method shown in B. (D) The Pearson's colocalization coefficient (PCC) was calculated for the fluorescent signal of H2B and PKC-θ as measured by confocal laser scanning microscopy in non-stimulated (NS) Jurkat T cells, and cells after primary (1°) and secondary (2°) stimulations (mean±s.e.m., *n*=20). **P≤0.01, ****P≤0.0001 (Mann–Whitney test). (E) Representative immunoblot of phosphorylated H2B Ser32 and Ser36 in DMSO and 1 μM C27-treated Jurkat T cells with or without PMA and Ca^2+^ ionophore (PI) (*n*=3). (F) Normalized H2B Ser32p densitometry is shown for human CD4^+^ T cells treated with either the control siRNA (siCtrl) or PKC-θ siRNA (siPKC-θ) (mean±s.e.m., *n*=3 individuals). ***P≤0.001 (two-tailed Student's *t*-test). (G) FAIRE chromatin accessibility shown for *IL2* and *TNF* in in non-stimulated (NS) Jurkat T cells, and cells after primary (1°) and secondary (2°) stimulations transfected with vector only (VO), wild-type PKC-θ plasmid (WT) or cytoplasmic-restricted PKC-θ mutant (NLS) plasmids. FAIRE chromatin accessibility is normalized to results for *GAPDH* (mean±s.e.m., *n*=3). ***P≤0.001 (two-way ANOVA). (H) FAIRE chromatin accessibility shown for *IL2* and other promoters in naïve and memory CD4^+^ T cells treated with either the control (siCtrl) or the PKC-θ siRNA (siPKC-θ) with or without PMA and Ca^2+^ ionophore (P/I). FAIRE chromatin accessibility is normalized to *GAPDH* and expressed as a percentage relative to the stimulated (ST) memory CD4^+^ T cells treated with the control siRNA (siCtrl) (mean±s.e.m., *n*=3). *P≤0.05, **P<0.01 (unpaired two-tailed Student's *t*-test).

Given that PKC-θ directly associates with the chromatin template, we used histone peptide microarrays to identify potential PKC-θ substrates. Significant levels of PKC-θ-mediated phosphorylation was detected on H2B- and H2A-derived peptides, particularly those derived from H2B, SKKGFKKAVVKTQKKEGKKR (H2B:11, amino acids 11–31 of the complete H2B sequence), and KTQKKEGKKRKRTRKESYSI (H2B:21, amino acids 21–40 of the complete H2B sequence). The top phosphorylation signal belonged to AQKKDG**RKRKRS**RKESYSVY (H2B:22, amino acids 22–41 of the complete H2B sequence) (Fig. 7A) containing the RxRxxS recognition pattern (bold) shared by many PKC-θ-targeted substrates such as BAD, NDRG and Rapgef2 (Hornbeck et al., 2012). Here, the highest signal was generated with arginine at residue 27 (Arg27), whereas replacement of alanine 21 with a valine residue substantially reduced phosphorylation to below background, suggesting that Arg27 contributes to optimal phosphorylation whereas Ala21 is crucial for substrate recognition (Fig. 7A). Substrate recognition was also heavily dependent on adjacent histone modifications. For example, butyrylation and propionylation at Lys21, Lys25, Lys28 and Lys29 generally promoted phosphorylation, but malonylation and succinylation at similar residues reduced phosphorylation. Furthermore, lysine methylation exerted status and positional effects on PKC-θ-mediated phosphorylation, such that monomethylation at lysine residues (e.g. Lys20, Lys21, Lys25, Lys28, Lys30, Lys32, Lys34 and Lys35) increased phosphorylation but trimethylated Lys21, Lys25, Lys28 and Lys34 discouraged phosphorylation (Fig. S4H).

Of the three serine residues on H2B:22, Ser32 appeared to be the important regulatory site, given that pre-phosphorylation of Ser32 significantly inhibited phosphorylation of Ser36 and Ser38 on variant H2B_FS (accession number P57053) and variants H2B_1, H2B_1K, H2B_1H, H2B_1M, H2B_1N, H2B_1D and H2B_1L (accession numbers P62807, O60814, Q93079, Q99879, Q99877, P58876 and Q99880, respectively; referred to as and H2B_1/1K/1H/1M/1N/1D/1L) (Fig. 7A). Based on X-ray crystallography of the nucleosome, Ser32 exists in a region known as the H2B repression domain (HBR), a DNA–histone interaction point (Lau et al., 2011). It is therefore reasonable to speculate that PKC-θ regulates gene expression by altering chromatin accessibility through H2B Ser32 phosphorylation in T cells. To further confirm PKC-θ phosphorylation specificity, we performed *in vitro* kinase assays using an enzymatically active PKC-θ with H2B, H3.1f and H4, or whole nucleosomes containing either the core histone H3.1 or the variant histone H3.3. Subsequent western blotting with anti-H2B-Ser32p and -Ser36p antibodies detected H2B-Ser32p and H2B-Ser36p signals only in the H2B histone but not H3.1 or H4. Similarly, H2B Ser32p and Ser36p were also evident in both H3.1- and H3.3-containing nucleosomes (Fig. 7B,C). Controls without the addition of ATP or PKC resulted in undetectable phosphorylation, reflecting the ATP-dependent PKC-θ catalytic activity. Similarly, incubation with PKC-µ also did not lead to H2B Ser32 or Ser36 phosphorylation, demonstrating the specificity of H2B Ser32 and Ser36 phosphorylation by PKC-θ. Therefore, PKC-θ can phosphorylate H2B Ser32 when it is present either as a peptide or in the context of a histone and a complete nucleosome. This association was further supported by confocal microscopy where the Pearson's colocalization co-efficient (PCC) between H2BSer32p and PKC-θ was significantly higher after the secondary activation than after the primary activation (Fig. 7D).

We further investigated this phosphorylation mechanism by pre-treating Jurkat T cells with C27. Without having any effect on Ser36p, C27 substantially prevented the increase of H2B Ser32p following activation compared to the control (Fig. 7E). As revealed by confocal microscopy, this lack of nuclear-tethered PKC-θ upon NLS mutation resulted in an overall loss of nuclear H2B Ser32p (Fig. S4I,J). In support of these data, a similar decrease in H2B Ser32p was also shown in the nuclear extract of PKC-θ knockdown human CD4^+^ T cells (Fig. 7F), demonstrating the dependence of H2B Ser32p on PKC-θ.

To investigate whether nuclear PKC-θ regulates chromatin accessibility through H2B, formaldehyde-assisted isolation of regulatory elements (FAIRE) was used to quantify chromatin accessibility across PKC-θ-targeted regions in Jurkat T cells. PMA and Ca^2+^ ionophore activation of vector only cells and cells expressing WT PKC-θ resulted in a highly accessible *IL2* promoter with increasing accessibility during re-stimulation. However, in the NLS-PKC mutant, *IL2* promoter accessibility was constrained by 58% in primary cells and 92% in secondary activated cells compared to the WT control. This NLS-PKC mutation also decreased the chromatin accessibility following activation at regions bound by PKC-θ at the *TNF*, *TNFSF9* and *SATB1* loci (Fig. 7G). To further investigate this, we carried out FAIRE analysis in the *ex vivo* memory CD4^+^ T cell model. Supporting the hypothesis that specific immune-regulatory genes progressively acquire chromatin accessibility during memory T cell development (Youngblood et al., 2013), there was a gradual gain in basal chromatin accessibility at the *IL2* promoter from naïve to memory T cells. Compared to *IL2*, *TNF* exhibited inducible chromatin accessibility in both activated naïve and memory CD4^+^ T cells. As a result of PKC-θ knockdown, chromatin accessibility for both of these inducible genes was significantly abrogated in activated memory CD4^+^ T cells compared to the siRNA control. Interestingly, the chromatin accessibility of *TNFSF9* and *SATB1* was inducible only in activated naïve CD4+ T cells but the lack of PKC-θ only restricted that of *TNFSF9* whereas *SATB1* remained affected. This chromatin profile of *SATB1* was similar to *CD69*, a non-PKC-θ-binding gene (Fig. 7H; Fig. S4K). Collectively, our data indicate that PKC-θ has the ability to specifically phosphorylate H2B Ser32 *in vitro* and *in vivo*. Furthermore, gene-specific analysis defines nuclear PKC-θ as a participant in configuring the chromatin accessibility of genes such as *IL2* and *TNF*, which is a prerequisite for inducible gene transcription in human memory CD4^+^ T cells.

## DISCUSSION

Memory T cells are poised for rapid transcriptional responses to antigenic re-stimulation. Understanding the molecular basis of transcriptional memory responses is key to vaccine design. Here, we determine that the signaling kinase PKC-θ, which relays incoming stimulatory signals directly to the permissive chromatin platform, plays a crucial role in eliciting immediate transcriptional memory responses in memory CD4^+^ T cells.

PKC-θ mediates signals from the co-stimulated T cell receptor (TCR) and is required for early survival of effector CD8^+^ T cells (Barouch-Bentov et al., 2005) and memory T cell development (Hollsberg et al., 1993; Teixeiro et al., 2009). We discovered that activation of Jurkat T cells with PMA (a PKC pathway inducer) has the ability to initiate transcriptional memory responses *in vitro*. Conversely, PKC-θ knockdown in *ex-vivo*-derived human memory CD4^+^ T cells disrupted the expression of pro-inflammatory genes and transcription factors that determines memory T cell quality (La Gruta et al., 2004; Seder et al., 2008), and effector and memory T cell differentiation (Thaventhiran et al., 2013).

In this study, PKC-θ knockdown or inhibition impairs transcriptional responses by reducing the activation of downstream pathways, such as NF-κB signaling. By using a promoter-biased ChIP-on-Chip approach, we previously reported on the overlap between NF-κB-binding sites and PKC-θ binding in Jurkat T cells (Sutcliffe et al., 2012). Here, we extended this investigation by using ChIP-seq and whole transcriptomic analysis in both *ex-vivo*-derived and Jurkat memory models to examine PKC-θ-decorated regions across the entire genome in naïve and memory human T cells. In light of this, we reproducibly identified a subset of transcriptional-memory-responsive genes that positively regulated NF-κB pathways *in vitro* and *ex vivo*. The promoters of genes highly expressed in vaccinia-responsive T_RM_ and T_EM_ cells were generally enriched for NF-κB family motifs. NF-κB p65 nuclear translocation and gene-specific recruitment were preferentially higher in re-stimulated Jurkat T cells and primary human memory CD4^+^ T cells activated *in vitro*. Increased TCR–NF-κB activity has previously been attributed to accelerated memory T cell responsiveness (Paul and Schaefer, 2013). In a process independent of TCR engagement, OX40 co-stimulation has been reported to activate NF-κB by forming a signalosome with PKC-θ, TRAF2, RIP2, IKKα, β and γ, and the CBM complex (So and Croft, 2012; So et al., 2011). Co-expression of OX40 and CD25 is associated with cytomegalovirus (CMV)-specific CD4^+^ T cell antigen-recall responses in healthy and chronic HIV^+^ patients (Phetsouphanh et al., 2014). Regardless of whether PKC-θ is activated by the TCR or OX40 signaling, our data indicate that enhanced PKC-θ activity and NF-κB upregulation act as a positive-feedback circuit that drives rapid transcriptional memory responses.

Genome occupancy of protein kinases such as mitogen-activated protein kinases (MAPKs) regulates transcription by association with transcription factors, chromatin-modifying enzymes and nucleosomal components in lower eukaryotes (Pokholok et al., 2006). Our study using human memory CD4^+^ T cells showed that PKC-θ localizes to permissive epigenetic regions marked by H3K4me3 and H3K9ac, where its catalytic activity is essential for p65 Ser536 phosphorylation. This is a novel mechanism of NF-κB target gene regulation in a stimulation-dependent manner, and reflects that nuclear-tethered PKC-θ is more than just a signal transducer and also acts as an intermediary between stimulatory signals and chromatin modifications, providing memory T cells with their cardinal feature of an immediate transcriptional response to re-infection. Given the broad involvement of signal transduction kinases in many physiological processes, it remains to be seen whether this is a more general gene regulatory mechanism.

Our genome-wide analysis demonstrated that PKC-θ largely binds to quiescent genomic regions that lack histone modifications and regulatory domains including intronic enhancers. Similarly, re-stimulation of Jurkat T cells directly targeted PKC-θ to H3K4me1- and H3K27ac-enriched regions, which demarcate lineage-specifying enhancers in human CD4^+^ T cell subsets (Schmidl et al., 2014). The presence of nuclear-tethered PKC-θ at these regions indicates that it might assume a structural or regulatory role to maintain enhancer interactions or remodel chromatin, which potentially explain the differences in PKC-θ binding observed between memory T cell subsets.

Although PKC family members are known to phosphorylate histone substrates, our results suggest that PKC-θ catalytic activity facilitates chromatin accessibility through core histone H2B phosphorylation. Previously, phosphorylation of Ser32, Ser36 and Ser38 have been shown to participate in mitogenic responses, transcription and survival (Bungard et al., 2010; Lau et al., 2011; Takai et al., 1977). Ser32 appears to be a major regulatory site, as its phosphorylation or repositioning can dramatically change chromatin configuration, in turn altering Ser36 and Ser38 accessibility and their capacity for phosphorylation. Ser36 and Ser38 are located at the start of the N-terminal helix, whereas Ser32 forms part of the N-terminal tail, with a protruding hydroxyl side chain facing the DNA phosphate backbone. Together with the two N-terminal acidic side chain residues (Arg31 and Lys30) in close proximity to DNA, Ser32 is predicted to be highly constrained and able to interact with DNA with high affinity (Tachiwana et al., 2010). Subsequent histone modifications to neighboring lysine residues might influence Ser32 recognition by PKC-θ. Given chromatin experiences rapid H2B substitution (Jamai et al., 2007), our results indicate that aberrant H2B phosphorylation in NLS-PKC mutant cells alters the degree of chromatin accessibility during primary and secondary activation. This implies that one role of PKC-θ is to maintain permissive chromatin, particularly in the context of T cell activation where chromatinized PKC-θ is required for H2B Ser32 phosphorylation. Together with other histones, H2B facilitates the chromatin accessibility necessary for the binding of transcription factors such as p65.

In summary, we show that nuclear localization of PKC-θ integrates activating signals at the chromatin template. This process facilitates rapid transcriptional programs in memory CD4^+^ T cells by maintaining p65 binding and chromatin accessibility through H2B Ser32 phosphorylation in a stimulation-dependent manner.

## MATERIALS AND METHODS

### Cell culture

The human Jurkat T cell line (Clone E6-1, ATCC TIB-152) was activated at 5×10^5^ cells/ml with 24 ng/ml phorbol 12-myristate 13-acetate (PMA; Sigma-Aldrich, P8139) and 1 µM Ca^2+^ ionophore (Sigma-Aldrich, A23187). The stimulus was withdrawn by washing three times with stimulus-free medium.

### Primary cells and flow cytometry

Primary human CD4^+^ T cells were isolated from buffy coats obtained from the Australian Red Cross Blood Service, Sydney. Peripheral blood mononuclear cells (PBMCs) were isolated using Ficoll-Hypaque density centrifugation (GE Healthcare, 17-1440-03). Total CD4^+^ T cells were negatively selected (Invitrogen, 113.46D). Memory CD4^+^ T cell subsets were sorted as previously described (Seddiki et al., 2006). Day 13 PBMCs were also isolated from healthy individuals undergoing routine primary inoculation with vaccinia virus and sorted into naïve (CD45RO^−^ CD38^lo^), resting memory (CD45RO^+^ CD38^−^), and activated (CD45RO^+^ CD38^+^) CD4^+^ T cell subsets. All human samples were used in accordance with University Ethics Committee guidelines (Ethics Committee code EC00140). The FoxP3 permeabilization solution kit (BioLegend) was used for intracellular staining with phycoerythrin-conjugated anti-p50 or anti-p65 antibodies (Abcam), detected using an LSRII flow cytometer (BD), and analyzed with FlowJo version 7.6.5.

### PKC-θ-specific siRNA and transfections

Human naïve or memory CD4^+^ T cells were transfected for 48 h with PKC-θ (sc-36252, Santa Cruz Biotechnology) and FAM-labeled mock control siRNAs together or FAM-labeled negative control siRNA alone in Lipofectamine 2000 and Opti-MEM. Jurkat T cells were transfected with 5 μg of vector-only plasmid or HA-tagged wild-type PKC-θ or cytoplasm-restricted PKC-θ plasmids using the NEON™ Transfection System kit (Invitrogen, MPK5000).

### Western blotting

Immunoblotting of whole Jurkat T cell lysates or nuclear extracts (Sutcliffe et al., 2012) was performed with: anti-PKC-θ (sc-212, Santa Cruz Biotechnology), anti-PKC-θ S676p (ab47774, Abcam), p65 (ab7970, Abcam), H3 (ab1791), Sp-1 (sc-59, Santa Cruz Biotechnology), p50 (sc-1191, Santa Cruz Biotechnology), p65 Ser486p (3039, Cell Signaling Technology), p65 Ser536p (3031, Cell Signaling Technology), IκB-α (Ser32/36; 9246, Cell Signaling Technology), H2B Ser32p (ab10476, Abcam), and H2B Ser36p (ECM Biosciences HP4331) antibodies with a dilution range of 1:200 to 1:1000. Signals were detected by enhanced chemiluminescence on a LAS4000 Fluoimager.

### NF-κB and kinase activity assays

The TransAM^®^ NFκB Transcription Factor ELISA kit (Active Motif, 43296) was used to quantify NF-κB activation in nuclear extracts. Recombinant PKC-θ (Invitrogen, PV3605) treated with 1 μM C27 was used, and activity was measured with a PKC Kinase Activity kit (Enzo ADI-EKS-420A).

### *In vitro* kinase assay

A mixture of kinase reaction buffer (ADI-EKS-420A, ENZO Life Sciences) containing 25 µM ATP and 5 µg recombinant histone was pre-warmed for 10 min at 30°C. Recombinant histone H2B (31252), H3.1 (31294), nucleosome H3.1 (31466), nucleosome H3.3 (31468) and histone H4 (31223) were used (Active Motif). The reaction was initiated by adding 20 ng recombinant PKC-θ (PV3605, Life Technologies) or PKC-μ (ADI-EKS-420A, Enzo Life Sciences) with the final reaction mixture (40 µl) incubated for 30 min at 30°C. The resulting phosphorylated proteins were resolved on 4–20% SDS-polyacrylamide gels followed by western blotting.

### Kinase profiling

JPT Peptides Technologies (Berlin, Germany) performed kinase profiling on peptide microarrays using active recombinant PKC-θ. Experiments were performed using a 20-meric unmodified or modified human histone peptide library: 604 H1 peptides (seven subtypes), 1093 H2A peptides (15 subtypes), 978 H2B peptides (15 subtypes), 922 H3 peptides (five subtypes) and 271 H4 peptides (one subtype) to include all known and synthetically accessible natural variants. Acetylation (lysine, KAc), methylation [lysine, Kme1, Kme2, Kme3; arginine, Rme1, Rme2a (asymmetric), Rme2s (symmetric)], butyrylation (lysine, KBut), propionylation (lysine, KProp), malonylation (lysine, KMal), succinylation (lysine, KSuc), citrullination (arginine, Cit) and phosphorylation (threonine, pT; serine, pS; tyrosine, pY) were considered.

Kinase was added to the peptide microarray in the presence of γ-[^33^P]-ATP and subsequently phosphoimaged to detect ^33^P incorporation. The mean signal intensities after image conversion ranged between ∼49,800 and ∼65,400 (median ∼54,400 units). An arbitrary threshold of 57,500 was determined to distinguish signal from background. Microarrays were repeated with the PKC-θ inhibitor C27 as a negative control, resulting in a significant signal decrease to 32,500. All peptides were immobilized on three identical spots per subarray, yielding nine instances of each peptide and results are given as mean±s.d. Given that each peptide was deposited in three adjacent spots, artifacts were easily detected. Asymmetric spots in the vicinity of very strong signals were discounted. GenepixPro and ArrayPro 4.0 were used for spot recognition and data analysis. Excel, R and Python custom scripts (available on request) were used for statistical analysis.

### Confocal microscopy

Jurkat T cells overexpressing vector-only, wild-type PKC-θ or PKC-NLS plasmids were probed with rabbit anti-p65 (Abcam ab7970) or anti-H2B (Abcam, ab10476) primary antibodies (1:100 to 1:200 dilution) and visualized with Alexa-Fluor 488-conjugated goat-anti-rabbit-IgG secondary antibodies (1:1000 dilution; Life Technologies, A11008). Nuclear p65 and H2B fluorescence was detected by confocal laser scanning microscopy. For each sample, Fiji-ImageJ software was utilized to determine Fn/Fc values which were calculated by: Fn/c=(Fn−Fb)/(Fc−Fb), where Fn is nuclear fluorescence, Fc is cytoplasmic fluorescence, and Fb is background fluorescence. Data represent the mean±s.e.m. Channels were overlaid to examine co-localization of the antibody targets. The Pearson's co-localization coefficient (PCC) was calculated with Fiji-ImageJ.

### RNA extraction and qPCR

Total mRNA was extracted using TRIzol (Invitrogen, 15596-018) followed by chloroform extraction (Sigma, C2432) and precipitation was achieved with isopropanol and converted into cDNA using the SuperScript™ III First-Strand Synthesis System (Invitrogen, 18080-051). Gene expression was determined by qRT-PCR with gene-specific TaqMan probes or TaqMan Low Density Arrays. Experiments were performed in triplicate unless otherwise stated.

### Chromatin immunoprecipitation

ChIP-qPCR was performed as outlined in Sutcliffe et al., 2011. ChIP-DNA was quantified by qRT-PCR with the primer sets listed in Table S6. ChIP-DNA was used to prepare ChIP-seq DNA libraries using the NEBNext^®^ ChIP-seq Library Prep Master Mix for Illumina^®^ (New England BioLabs Inc., E6240L). ChIP DNA libraries were sequenced on the Illumina HiSeq2000 using single-read 50-bp runs.

### Microarray analysis

Affymetrix GeneChip^®^ Hugene 1 (Jurkat) or Human Genome U133 Plus 2.0 Arrays (vaccinia) were used for transcriptomics. Affymetrix microarray data including E-MEXP-2578 was robust multi-array average (RMA) normalized in affy (Gautier et al., 2004). Pearson correlations were calculated for all peaks and main array probes and visualized using gglot2 and reshape 2. Overexpression was defined as a log_2_0.5 increase. Gene set enrichment analysis (GSEA) was used with default parameters using gene set permutations and differences of classes with a ≤25% false discovery rate (FDR) and a nominal *P*-value of ≤0.05.

### Bioinformatics analysis

Sequences were trimmed (Cutadapt) and mapped to the human genome (Hg19) using local alignment in Bowtie 2 (Langmead and Salzberg, 2012). Duplicate reads were removed (Picard) and enriched regions were called against the relevant total input using a 0.01 q-value cut-off and the broad peak option in MACS2 (Zhang et al., 2008). A cut-off of ≥175 bp for the primary regions was used to remove low complexity reads. The number of reads was counted and minus-average normalized in R software. Minus-average normalization was performed on the non-log counts using the R MASS and affy packages and adjusting counts using the coefficient from a linear model of the differences in counts and the count average. The code was similar to MAnorm (Shao et al., 2012), except that non-logged values were used. The nearest ENSEMBL transcript to the peaks was determined using ChIPpeakAnno (Zhu et al., 2010) and HOMER (Heinz et al., 2010), and classified as 5′UTR, exon, intron, promoter (−1 kb from the TSS), upstream (−10 kb to −1 kb from the TSS) or intergenic. Peaks were also annotated to their chromatin marks using Roadmap ENCODE data (Hoffman et al., 2013). NFKB ChIP-seq data were from GEO GSM935282. Heatmaps were created using HOMER and Heatplus. Transcription-factor-binding motifs were analyzed using HOMER (mask –200, *P*<10^−12^) (Heinz et al., 2010) and ISMARA (*z*-score cut-off of 2) (Balwierz et al., 2014) and CLOVER. Overlapping peaks were calculated in a similar way to read counting, but peaks were first merged and the original peak files treated as reads (with no extension step). CEAS was used to investigate the conservation scores of the PKC-θ-bound regions (Ji et al., 2006).
